# Supplementary material for: A Likelihood Approach for Real-Time Calibration of Stochastic Compartmental Epidemic Models
Source: PLoS Comput Biol. 2017 Jan 17;13(1):e1005257. doi: 10.1371/journal.pcbi.1005257 (PMC5240920; doi:10.1371/journal.pcbi.1005257)
Supplement: S1 File — (TAR.GZ) [file pcbi.1005257.s014.tar.gz › HSPH_Online-SI-Revision/EnKF07/n100-severe/n100-severe_PF_EnKF-summery.pdf]

peak -8	R0	$1/\theta_2$	inf	inflog	newly	newlylog	Reff	AttackRate
mean	62.9	83.6	46 916.4	1.9	24 582.1	1.6	56.2	143.5
median	61.7	58.2	21 252.2	2.2	11 449.8	1.8	57.	143.2
Quantile .1	53.6	38.3	343.2	0.5	237.3	0.4	34.9	86.3
Quantile .9	76.2	184.2	136 534.	3.	48 773.8	2.4	77.1	198.4
38	46	8						
length	50							

peak -4	R0	$1/\theta_2$	inf	inflog	newly	newlylog	Reff	AttackRate
mean	66.3	67.2	713.	0.6	291.1	0.6	68.7	178.3
median	64.4	55.1	147.2	0.5	157.7	0.6	68.5	172.6
Quantile .1	53.6	30.3	58.9	0.3	96.9	0.4	56.4	140.5
Quantile .9	85.2	115.3	2591.2	1.2	725.3	0.8	79.6	223.6
31	39	0						
length	50							

peak	R0	$1/\theta_2$	inf	inflog	newly	newlylog	Reff	AttackRate
mean	72.1	88.7	85.1	0.7	93.	0.8	54.6	186.7
median	71.9	79.6	83.7	0.6	90.8	0.9	54.6	178.2
Quantile .1	56.2	33.8	70.2	0.3	79.4	0.5	43.4	161.9
Quantile .9	89.4	139.3	101.6	1.	101.5	1.1	65.3	227.4
36	44	0						
length	50							

peak +4	R0	$1/\theta_2$	inf	inflog	newly	newlylog	Reff	AttackRate
mean	67.9	72.2	439.4	0.5	472.9	0.6	52.6	190.3
median	67.9	62.4	224.3	0.4	289.7	0.5	50.9	184.6
Quantile .1	52.3	21.	98.9	0.3	103.1	0.4	37.3	153.8
Quantile .9	86.1	131.1	909.3	0.8	988.8	0.8	71.9	223.4
27	32	0						
length	50							

peak +8	R0	$1/\theta_2$	inf	inflog	newly	newlylog	Reff	AttackRate
mean	70.3	103.6	17 122.7	1.3	10 416.4	1.2	61.5	186.9
median	67.4	88.4	2814.9	1.3	1815.9	1.	61.8	182.8
Quantile .1	57.	59.7	254.6	0.4	451.6	0.6	41.6	149.3
Quantile .9	85.4	178.4	31 768.	2.4	24 568.4	2.2	85.1	225.5
31	24	0						
length	50							
